# *In vitro* activities of zosurabalpin and comparator antimicrobials against clinical isolates of carbapenem-resistant *Acinetobacter baumannii*

**DOI:** 10.1128/spectrum.00318-26

**Published:** 2026-09-29

**Authors:** Yu-Tzu Lin, Hsiu-Hsien Lin, Sin-Rong Li, Kun-Hao Tseng, Zi-Lun Lai, Mao-Wang Ho, Po-Ren Hsueh

**Affiliations:** 1 Department of Medical Laboratory Science and Biotechnology, China Medical University 38020, Taichung, Taiwan; 2 Department of Laboratory Medicine, China Medical University Hospital, China Medical University 38019, Taichung, Taiwan; 3 Division of Infectious Diseases, Department of Internal Medicine, China Medical University Hospital, China Medical University 38020, Taichung, Taiwan; University of Guelph College of Biological Science, Guelph, Ontario, Canada

**Keywords:** zosurabalpin, carbapenem-resistant *Acinetobacter baumannii*, cefiderocol, colistin

## Abstract

Carbapenem-resistant *Acinetobacter baumannii* (CRAB) poses a major therapeutic challenge due to the limited availability of effective treatment options and is associated with substantially increased mortality in healthcare-associated infections. Zosurabalpin (ZAB, RG6006) is a novel macrocyclic peptide (MCP) antibiotic that exerts its antibacterial activity by inhibiting lipopolysaccharide transport, ultimately leading to bacterial cell death. This study evaluated the *in vitro* activities of ZAB and other antimicrobial agents against clinical CRAB isolates. A total of 206 clinical *A. baumannii* isolates were collected, including 189 CRAB and 17 carbapenem-susceptible *A. baumannii* (CSAB). Minimum inhibitory concentrations (MICs) of ZAB were determined using broth microdilution in cation-adjusted Mueller–Hinton broth supplemented with 20% heat-inactivated horse serum. MICs of 11 other comparator agents, including cefiderocol (FDC) and colistin (COL), were determined following Clinical and Laboratory Standards Institute (CLSI) guidelines. *A. baumannii* NCTC 13304 was used as the quality control strain for zosurabalpin. MIC values for *A. baumannii* NCTC 13304 tested with ZAB ranged from 0.03 to 0.06 µg/mL (reference range: 0.016–0.12 µg/mL). ZAB demonstrated potent *in vitro* activity against CRAB, with MIC_50_ and MIC_90_ values of 0.25 and 1 μg/mL, respectively. Among FDC-resistant CRAB isolates, the MIC_50_/MIC_90_ remained 0.25/1 μg/mL, and for COL-resistant CRAB isolates, the MIC_50_/MIC_90_ was 0.5/2 μg/mL. Notably, a CRAB isolate, which was resistant to both FDC and COL, exhibited a ZAB MIC of 0.5 μg/mL. This study demonstrated that ZAB exhibited potent *in vitro* activity against CRAB, independent of resistance to FDC and/or COL. These findings support ZAB as a promising therapeutic candidate for difficult-to-treat CRAB infections.

**IMPORTANCE:**

Carbapenem-resistant *Acinetobacter baumannii* (CRAB) remains a major challenge in clinical practice because of limited treatment options. Zosurabalpin (ZAB) is a first-in-class macrocyclic peptide antibiotic that has been proposed as a potential therapeutic agent for CRAB infections; however, data on its *in vitro* activity against clinical isolates remain limited. This study provides comprehensive *in vitro* susceptibility data for ZAB against clinical CRAB isolates, including strains resistant to colistin and cefiderocol. Our findings demonstrate that ZAB retains *in vitro* activity against CRAB, including isolates resistant to colistin and/or cefiderocol, and support its potential as a promising therapeutic option for the treatment of difficult-to-treat CRAB infections.

## INTRODUCTION

The *Acinetobacter calcoaceticus–Acinetobacter baumannii* (ACB) complex consists of six species: *Acinetobacter baumannii*, *Acinetobacter calcoaceticus*, *Acinetobacter nosocomialis*, *Acinetobacter pittii*, *Acinetobacter seifertii*, and *Acinetobacter lactucae*. *A. baumannii* is the most clinically significant of these species and frequently related to healthcare-associated infections, causing severe diseases, such as hospital-acquired pneumonia and bloodstream and urinary tract infections (1). Because of its remarkable ability to acquire exogenous resistance determinants, the susceptibility of *A. baumannii* to multiple antimicrobial agents has progressively decreased (2). Clinically, the proportion of carbapenem-resistant *A. baumannii* (CRAB), including extensively drug-resistant (XDR) and pandrug-resistant (PDR) strains, continues to rise, with carbapenem resistance exceeding 90% in some countries (3, 4).

The emergence of CRAB has made clinical management increasingly challenging. Compared with drug-susceptible *Acinetobacter* infections, CRAB and PDRAB are associated with markedly higher mortality rates (52 and 75%, respectively) (5). The World Health Organization (WHO) has designated CRAB as a critical-priority pathogen in the Bacterial Priority Pathogens List, underscoring the urgent need for new therapeutic options against CRAB infections (6).

Due to the limited treatment options for CRAB infections, colistin (COL) has re-emerged as a critical last-line therapeutic agent (7). The drug had previously fallen out of use because of its well-recognized risks of neurotoxicity and nephrotoxicity (8). Although COL exhibits strong *in vitro* activity against CRAB, its narrow therapeutic window and toxicity profile may contribute to suboptimal clinical outcomes (8). The emergence of COL-resistant isolates has further exacerbated this challenge (9).

Several new antibiotics have been developed in recent years, and some have received United States (U.S.) Food and Drug Administration (FDA) approval, including eravacycline and cefiderocol (FDC). FDC is the first catechol-substituted cephalosporin approved for the treatment of complicated urinary tract infections as well as hospital-acquired and ventilator-associated bacterial pneumonia (10, 11). However, certain β-lactamases can partially hydrolyze FDC, and its activity may be further reduced when β-lactamases coexist with other resistance mechanisms (12, 13). In addition, FDC resistance mediated by target-site mutations has also been reported (14).

Zosurabalpin (ZAB, RG6006) is a first-in-class macrocyclic peptide (MCP) antibiotic that targets the LptB_2_FGC complex located on the inner membrane surface (15, 16). The Lpt system is a key ABC transporter responsible for lipopolysaccharide (LPS) translocation. LPS transport involves the inner-membrane LptB_2_FGC complex, the periplasmic protein LptA, and the outer-membrane LptDE complex (17). LptB_2_FGC extracts LPS from the inner membrane and transfers it through a transport bridge formed by LptC, LptA, and LptDE, ultimately delivering LPS to the outer leaflet of the outer membrane (18, 19). When the transmembrane helix of LptC dissociates from the LptB_2_FGC complex, ZAB can bind within the LptB_2_FG transporter and arrest the transport cycle (16). This interaction stabilizes a stalled LPS–transporter intermediate, thereby preventing the progression of LPS transport from the inner to the outer membrane. This blockade leads to lethal intracellular accumulation of LPS, ultimately resulting in cell lysis.

To evaluate the *in vitro* activity of ZAB against clinical isolates, we collected ACB complex isolates from clinical specimens and determined their ZAB susceptibility. We then assessed its *in vitro* activity across different resistance phenotypes and compared its activity with that of other antimicrobial agents.

## MATERIALS AND METHODS

### Bacterial isolates

A total of 206 *A. baumannii* isolates were randomly recovered from the Microbiology Laboratory at China Medical University Hospital (CMUH) between January 2017 and February 2025 (Table 1). These isolates were recovered from various sources of patients, including respiratory tract (*n* = 80), blood (*n* = 61), body fluid (*n* = 27), pus/wound (*n* = 24), urine (*n* = 12), and catheter tips (*n* = 2) of patients treated at the hospital (Table 1). An additional 24 isolates from other members of the ACB complex were also included, consisting of 16 *A. nosocomialis* (six carbapenem-resistant [CR], 10 carbapenem-susceptible [CS]), five *A. seifertii* (four CR, one CS), two *A. pittii* (one CR, one CS), and one *A. lactucae* (CS). All these isolates were identified using a Matrix-Assisted Laser Desorption/Ionization Time-of-Flight Mass Spectrometry (MALDI-TOF MS) Biotyper System (Bruker Microflex LT/SH, Bruker Daltonics GmbH, Bremen, Germany). Carbapenem susceptibility testing was performed using the BD Phoenix Automated System (Becton-Dickinson Microbiology Systems, Sparks, MD, USA). The isolates with imipenem and meropenem minimum inhibitory concentrations (MICs) ≥ 4 µg/mL were defined as CR as per Clinical and Laboratory Standards Institute (CLSI) guidelines. Imipenem and meropenem susceptibilities were also assessed using the disk diffusion method according to CLSI guidelines (20). Bacterial suspensions were adjusted to a 0.5 McFarland standard and inoculated onto Mueller–Hinton agar plates. Imipenem (10 µg) and meropenem (10 µg) BD BBL Sensi-Disc disks (Becton-Dickinson, Sparks, MD, USA) were applied, and plates were incubated at 35°C for 20–24 h. *Escherichia coli* ATCC 25922 was used as the quality control strain.

**TABLE 1:** A total of 230 isolates of *Acinetobacter calcoaceticus–Acinetobacter baumannii* (ACB) complex collected from various patients’ sources were evaluated in this study

Species (no. of isolates)	Carbapenem-resistant		Carbapenem-susceptible	
	No. of isolate	Source (no.)	No. of isolate	Source (no.)
*Acinetobacter baumannii* (206)	189	Respiratory (80), blood (44), body fluid (27), pus/wound (24), urine (12), catheter tip (2)	17	Blood (17)
*Acinetobacter nosocomialis* (16)	6	Respiratory tract (1), blood (4), pus/wound (1)	10	Blood (10)
*Acinetobacter seifertii* (5)	4	Respiratory tract (1), blood (1), body fluid (2)	1	Blood (1)
*Acinetobacter pittii* (2)	1	Respiratory tract (1)	1	Blood (1)
*Acinetobacter lactucae* (1)	0	N/A^*a*^	1	Blood (1)
Total (230)	200		30	

^*a*^ N/A, not applicable (no samples were available).

### MICs of ZAB

The standard powder of ZAB was obtained from Roche Pharma Research and Early Development (Basel, Switzerland). The MICs of ZAB were determined using the broth microdilution (BMD) method with cation-adjusted Mueller–Hinton broth (CAMHB) supplemented with 20% heat-inactivated horse serum according to Clinical and Laboratory Standards Institute (CLSI) guidelines (CLSI M07 and M100 documents) (20, 21). The concentration of ZAB tested ranged from 0.001 to 64 µg/mL. Bacterial inocula were first adjusted to a 0.5 McFarland standard in CAMHB. Serial twofold dilutions of ZAB were prepared in 96-well microtiter plates, which were then inoculated with an appropriate volume of the standardized bacterial suspension to achieve a final inoculum of approximately 5 × 10^5^ CFU/mL. The plates were incubated at 35°C for 20–24 h. *A. baumannii* NCTC 13304 and *A. baumannii* ATCC 15091 were used as the quality control (QC) strains.

### MICs of FDC

The MICs of FDC were determined using the BMD method with the Shionogi MIC Dry Plate Cefiderocol (Shionogi & Co., Ltd., Osaka, Japan) (22). Procedures were performed according to the manufacturer’s instructions and CLSI guidelines M07 and M100 (20, 21). FDC was tested in iron-depleted CAMHB containing twofold stepwise concentrations ranging from 32 to 0.03 µg/mL. The test inoculum was prepared by mixing 0.05 mL of a standardized culture with 12 mL of sterile distilled water, and 0.1 mL of this suspension was dispensed into each well of the microtiter plate. The plates were incubated at 35°C for 20–24 h. *E. coli* ATCC 25922 and *Pseudomonas aeruginosa* ATCC 27853 were included as QC strains.

### MICs of other comparator antimicrobial agents

The MICs of colistin (COL), imipenem, meropenem, ceftazidime/avibactam, ceftolozane/tazobactam, imipenem/relebactam, meropenem/vaborbactam, delafloxacin, eravacycline, omadacycline, plazomicin, and fosfomycin + glucose-6-phosphate against CRAB isolates were determined using the Sensititre MDRGNXXF plate (Thermo Fisher Scientific, Inc., Waltham, MA, USA). Testing was performed following the manufacturer’s protocol and the CLSI M07 and M100 guidelines (20, 21). A 50 µL aliquot of the inoculum adjusted to a 0.5 McFarland standard in CAMHB was added into each well of the test plate. MICs were read after incubation at 35°C for 20–24 h. *E. coli* ATCC 25922 was used as the QC strain according to the manufacturer’s recommendations.

### MIC_50_ and MIC_90_

MIC_50_ and MIC_90_ were calculated from the MIC distribution of each isolate group using the ordered-array method (23). Briefly, MIC values were arranged in ascending order, and MIC_50_ and MIC_90_ were defined as the MIC values at which at least 50 and 90% of the isolates were inhibited, respectively.

## RESULTS

### Activity of ZAB against CRAB and CSAB

**Fig 1:**
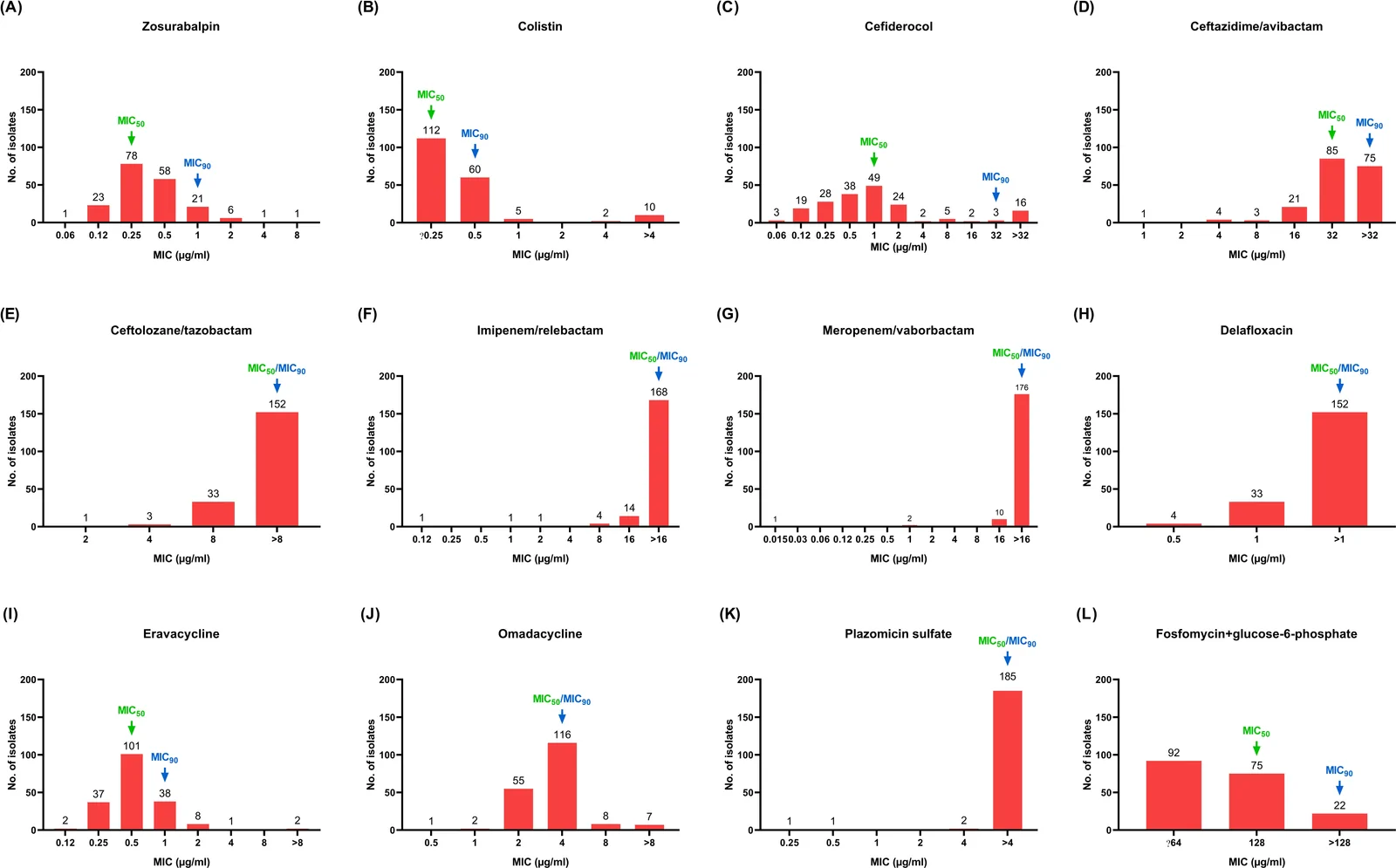
Distribution of zosurabalpin and other 11 antimicrobial agents’ MICs among carbapenem-resistant *Acinetobacter baumannii* (CRAB) isolates. (**A**) Zosurabalpin. (**B**) Colistin. (**C**) Cefiderocol. (**D**) Ceftazidime/avibactam. (**E**) Ceftolozane/tazobactam. (**F**) Imipenem/relebactam. (**G**) Meropenem/vaborbactam. (**H**) Delafloxacin. (**I**) Eravacycline. (**J**) Omadacycline. (**K**) Plazomicin. (**L**) Fosfomycin + glucose-6-phosphate. Red bars indicate isolate frequencies at each MIC value; MIC_50_ and MIC_90_ values are highlighted with green and blue arrows, respectively.

**Fig 2:**
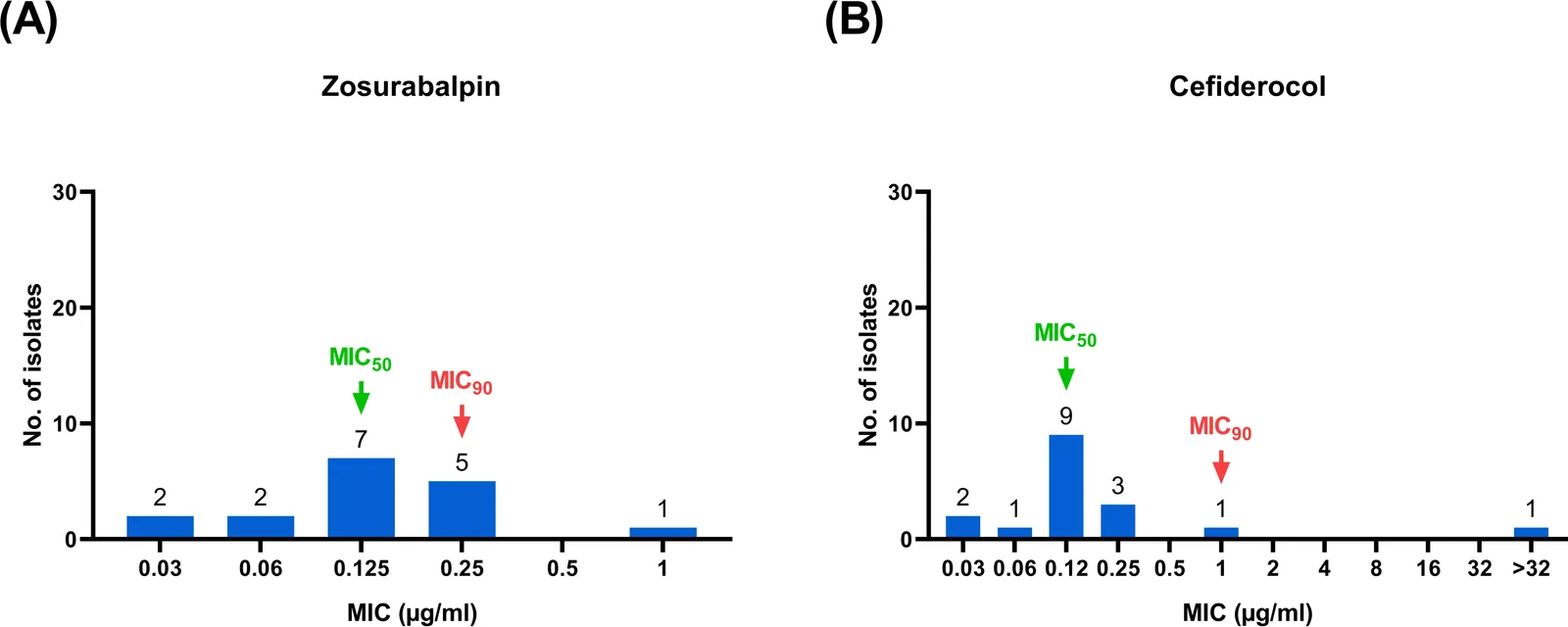
Distribution of zosurabalpin and cefiderocol MICs among carbapenem-susceptible *A. baumannii* (CSAB) isolates. (**A**) Zosurabalpin. (**B**) Cefiderocol. Blue bars indicate isolate frequencies at each MIC value; MIC_50_ and MIC_90_ values are highlighted with green and red arrows, respectively.

Susceptibility results for imipenem and meropenem among CRAB, CSAB, and other ACB complex isolates were consistent between the BD Phoenix Automated System and the disk diffusion method. ZAB MIC values against *A. baumannii* NCTC 13304 ranged from 0.03 to 0.06 µg/mL (reference range, 0.016–0.12 µg/mL). Against the 189 CRAB isolates, the MICs ranged from 0.06 to 8 μg/mL with MIC_50_ and MIC_90_ of 0.25 and 1 μg/mL, respectively (Table 2; Fig. 1). For eight (4.2%) CRAB isolates, the MIC values were ≥2 µg/mL. In contrast, against the 17 CSAB isolates, the MIC_50_ and MIC_90_ were 0.125 and 0.25 μg/mL, respectively, with MIC values ranging from 0.03 to 1 μg/mL (Table 2 and Fig. 2). Overall, the MIC_50_/MIC_90_ values of ZAB against CRAB isolates were twofold and fourfold higher, respectively, than those against CSAB isolates. Although MIC values were generally higher against CRAB isolates than CSAB isolates, only ZAB, COL, and eravacycline achieved MIC_90_ values of ≤1 µg/mL for both groups among the agents tested (Table 2; Fig. 1 and 2). These findings confirm that ZAB displays potent *in vitro* activity against both CRAB and CSAB. We also determined the ZAB MIC against *A. baumannii* ATCC 15091. Across eight independent tests, the MIC ranged from 0.25 to 0.5 μg/mL.

**TABLE 2:** *In vitro* susceptibilities of zosurabalpin and other 11 antimicrobial agents against 206 isolates of *A. baumannii* determined by the broth microdilution method categorized by carbapenem susceptibility of the isolates

Antibiotics	MIC (μg/mL)						
	Concentration range of tested agent	Carbapenem-resistant (*n* = 189)			Carbapenem-susceptible (*n* = 17)		
		Range	MIC_50_	MIC_90_	Range	MIC_50_	MIC_90_
Zosurabalpin	0.001 to 64	0.06 to 8	0.25	1	0.03 to 1	0.125	0.25
Polymyxins							
Colistin	0.25 to 4	≤0.25 to >4	≤0.25	0.5	≤0.25 to 4	≤0.25	0.5
Siderophore cephalosporin							
Cefiderocol	0.03 to 32	0.06 to >32	1	32	0.03 to >32	0.12	1
β-Lactam/β-lactamase inhibitors							
Ceftazidime/avibactam	0.12/4 to 32/4	1/4 to >32/4	32/4	>32/4	2/4 to >32/4	32/4	>32/4
Ceftolozane/tazobactam	0.06/4 to 8/4	2/4 to >8/4	>8/4	>8/4	≤0.06/4 to >8/4	4/4	>8/4
Imipenem/relebactam	0.03/4 to 16/4	0.12/4 to >16/4	>16/4	>16/4	0.12/4 to > 16/4	1/4	>16/4
Meropenem/vaborbactam	0.008/8 to 16/8	0.015/8 to > 16/8	>16/8	>16/8	0.12/8 to > 16/8	0.5/8	>16/8
Fluoroquinolones							
Delafloxacin	0.12 to 1	0.5 to > 1	>1	>1	≤0.12 to >1	≤0.12	>1
Tetracyclines							
Eravacycline	0.03 to 8	0.12 to > 8	0.5	1	≤0.03 to 1	0.06	0.5
Omadacycline	0.12 to 8	0.5 to > 8	4	4	≤0.12 to 8	0.25	4
Aminoglycosides							
Plazomicin	0.12 to 4	0.25 to > 4	>4	>4	0.5 to > 4	2	>4
Fosfomycin + glucose-6-phosphate	64 to 128	≤64 to >128	128	>128	≤64 to >128	128	>128

### Susceptibility of COL among CRAB

Among CRAB isolates, 177 (93.7%) isolates were intermediate (MICs, ≤2 µg/mL) to COL, and 12 (6.3%) were resistant (MICs ≥ 4 µg/mL) to COL (Table 3 and Fig. 1). Overall, COL exhibited the lowest MIC_90_ against CRAB (0.5 μg/mL) among all agents tested (Table 2). Notably, among the 12 COL-resistant CRAB isolates, the MIC_50_/MIC_90_ of ZAB was 0.5/2 μg/mL, respectively, and 83.3% (10/12) isolates were inhibited by ZAB at ≤1 µg/mL (Tables 3 and 4).

**TABLE 3:** MICs of zosurabalpin against *A. baumannii* isolates (*n* = 206) categorized by their resistance profiles to cefiderocol (FDC) and colistin (COL)

Resistance profile (no. of isolates)^*a*^	Zosurabalpin MIC (μg/mL)^*b*^		
	Range	MIC_50_	MIC_90_
CS (*n* = 17)	0.03–1	0.125	0.25
CR (*n* = 189)	0.06–8	0.25	1
FDC-S (*n* = 168)	0.125–4	0.25	1
FDC-R (*n* = 21)	0.06–8	0.25	1
COL-I (*n* = 177)	0.06–8	0.25	1
COL-R (*n* = 12)	0.125–2	0.5	2
FDC-R and COL-R (*n* = 1)	0.5	–	–

^*a*^ CS, carbapenem-susceptible; CR, carbapenem-resistant; S, susceptible; I, intermediate; R, resistant.

^*b*^ –, not determined due to insufficient sample size (*n* = 1).

**TABLE 4:** *In vitro* susceptibilities of zosurabalpin and other 11 antimicrobial agents against 12 carbapenem- and colistin-resistant *A. baumannii* isolates determined by the broth microdilution method

Isolates	MIC (μg/mL)											
	Colistin	Zosurabalpin	Cefiderocol	Ceftazidime/avibactam	Ceftolozane/tazobactam	Imipenem/relebactam	Meropenem/vaborbactam	Delafloxacin	Eravacycline	Omadacycline	Plazomicin	Fosfomycin + glucose-6-phosphate
CR-17	4	0.5	>32	>32/4	>8/4	>16/4	>16/8	>1	0.5	4	>4	128
CR-19	>4	2	1	>32/4	>8/4	>16/4	>16/8	>1	0.5	4	>4	128
CR-32	4	0.5	0.25	32/4	8/4	>16/4	>16/8	>1	1	4	>4	128
CR-38	>4	0.25	0.12	>32/4	>8/4	>16/4	>16/8	1	2	8	>4	>128
CR-40	>4	1	2	8/4	8/4	8/4	>16/8	0.5	0.25	8	>4	>128
CR-44	>4	0.5	0.25	4/4	4/4	16/4	16/8	1	0.25	2	4	>128
CR-48	>4	0.5	0.06	4/4	2/4	8/4	>16/8	1	0.5	4	4	>128
CR-52	>4	0.25	0.5	>32/4	>8/4	>16/4	>16/8	1	0.5	2	>4	128
CR-53	>4	0.5	0.12	8/4	4/4	8/4	>16/8	1	0.25	2	>4	>128
CR-97	>4	2	0.25	16/4	8/4	16/4	16/8	>1	1	4	>4	≤64
CR-126	>4	0.5	0.06	32/4	>8/4	16/4	16/8	>1	0.5	2	>4	≤64
CR-175	>4	0.125	1	>32/4	>8/4	>16/4	>16/8	>1	1	4	>4	>128

### Activity of FDC against CRAB and CSAB

The MIC values of FDC against the two QC strains *E. coli* ATCC 25922 and *P. aeruginosa* ATCC 27853 were 0.12 and 0.25–0.5 μg/mL, respectively, which were within the corresponding reference ranges of 0.06–0.5 μg/mL for both strains. Compared with ZAB, FDC showed lower MIC_50_/MIC_90_ values against CSAB (0.12/1 μg/mL), but its activity against CRAB was markedly reduced, with an MIC_90_ of 32 μg/mL (Table 2 and Fig. 1 and 2). Notably, 11.1% of CRAB isolates (21/189) were resistant to FDC (MIC ≥16 µg/mL), and 76.2% of these resistant isolates had MIC values exceeding 32 μg/mL. For the 21 FDC-resistant CRAB isolates, the MIC_50_ and MIC_90_ of ZAB were 0.25 and 1 μg/mL, respectively, and nearly all isolates (95.2%, 20/21) were inhibited by ZAB concentrations ≤ 1 µg/mL (Table 3). Importantly, for the CRAB isolate that was resistant to both FDC and COL, the MIC of ZAB was only 0.5 μg/mL (Table 3). Among CSAB isolates, most (93.8%, 15/16) remained susceptible to FDC; however, one isolate displayed an MIC of >32 µg/mL. In contrast, this isolate showed a ZAB MIC of only 0.125 μg/mL (data not shown).

### Activity of β-lactam and β-lactamase inhibitor combination agents against CRAB

MIC values of the QC strain *E. coli* ATCC 25922 for all the agents tested against CRAB and CSAB using Sensititre MDRGNXXF plate were all within the specified acceptable ranges. For CRAB, all four β-lactam/β-lactamase inhibitor combination agents (ceftazidime/avibactam, ceftolozane/tazobactam, imipenem/relebactam, and meropenem/vaborbactam) tested showed MIC_50_ and MIC_90_ values at the upper limit of the testing range (Table 2 and Fig. 1).

### Activity of delafloxacin against CRAB

Delafloxacin, a novel anionic, non-zwitterionic broad-spectrum fluoroquinolone, showed limited *in vitro* activity against CRAB isolates, with MIC_90_ values exceeding 1 µg/mL (Table 2; Fig. 1 and 2). The MIC_50_ for CRAB was also greater than 1 µg/mL. Interestingly, among the 12 COL-resistant CRAB isolates, half exhibited lower delafloxacin MICs (0.5–1 µg/mL) (Table 4).

### Activity of eravacycline and omadacycline against CRAB

Eravacycline, a new tetracycline-class antibiotic, demonstrated good activity against CRAB isolates. The MIC_90_ for CRAB was 1 μg/mL, ranking second only to colistin and comparable to that of ZAB (Table 2 and Fig. 1). Notably, two CRAB isolates exhibited elevated eravacycline MICs (>8 µg/mL) (Fig. 1). In contrast, the other new tetracycline, omadacycline, showed limited activity against CRAB, with an MIC_90_ of 4 μg/mL (Table 2). Omadacycline MICs were also elevated among the COL-resistant CRAB isolates, ranging from 2 to 8 μg/mL (Table 4).

### Activity of plazomicin against CRAB and CSAB

Plazomicin, a new aminoglycoside, showed poor *in vitro* activity against CRAB isolates. Among CRAB isolates, 97.9% (185/189) had MICs >4 µg/mL, and only two isolates exhibited MICs below 0.5 μg/mL (Fig. 1).

### MIC distribution of non-*baumannii* ACB complex isolates

In this study, we also included 24 non-*baumannii* ACB complex isolates (Table 1), with *A. nosocomialis* being the most common species (*n* = 16). Regardless of their carbapenem susceptibility status, ZAB showed consistently low MICs across these isolates, ranging from 0.015 to 1 μg/mL (Tables 5 and 6). Among the different species, *A. seifertii* exhibited a higher tendency toward COL nonsusceptibility, as half of the isolates, including both CR and CS isolates, had MICs of 4 μg/mL (Tables 5 and 6). In addition, FDC displayed elevated MICs among CR-*A. nosocomialis* and *A. seifertii* isolates, with ranges of 0.12–4 and 2–16 μg/mL, respectively (Table 5).

**TABLE 5:** *In vitro* susceptibilities of zosurabalpin and other 11 antimicrobial agents against 11 carbapenem-resistant non-*baumannii Acinetobacter calcoaceticus-Acinetobacter baumannii* complex isolates determined by the broth microdilution method

Isolates	MIC (μg/mL)											
	Zosurabalpin	Colistin	Cefiderocol	Ceftazidime/ avibactam	Ceftolozane/ tazobactam	Imipenem/ relebactam	Meropenem/ vaborbactam	Delafloxacin	Eravacycline	Omadacycline	Plazomicin	Fosfomycin + glucose-6-phosphate
Carbapenem-resistant *Acinetobacter nosocomialis* (*n* = 6)												
CR-59	0.125	0.5	4	>32/4	4/4	>16/4	>16/8	≤0.12	0.25	1	>4	≤64
CR-76	0.125	≤0.25	1	8/4	8/4	>16/4	>16/8	1	0.5	2	>4	≤64
CR-109	0.25	≤0.25	1	8/4	8/4	>16/4	>16/8	1	0.5	2	>4	≤64
CR-130	0.25	≤0.25	2	>32/4	>8/4	>16/4	>16/8	0.25	≤0.03	≤0.12	1	≤64
CR-174	0.25	1	1	>32/4	>8/4	>16/4	>16/8	>1	0.5	4	>4	≤64
CR-187	0.25	0.5	0.12	4/4	1/4	8/4	4/8	≤0.12	0.12	1	4	≤64
Carbapenem-resistant *Acinetobacter seifertii* (*n* = 4)												
CR-129	0.125	1	8	16/4	8/4	16/4	8/8	≤0.12	0.12	1	>4	≤64
CR-151	0.25	4	2	16/4	8/4	16/4	16/8	≤0.12	0.12	1	>4	128
CR-177	0.06	1	8	32/4	8/4	16/4	16/8	0.25	0.25	1	>4	≤64
CR-200	0.5	4	16	>32/4	>8/4	>16/4	>16/8	1	0.25	1	>4	≤64
Carbapenem-resistant *Acinetobacter pittii* (*n* = 1)												
CR-169	0.5	≤0.25	0.12	32/4	2/4	16/4	8/8	1	0.12	1	1	128

**TABLE 6:** *In vitro* susceptibilities of zosurabalpin and cefiderocol against 13 carbapenem-susceptible non-*baumannii Acinetobacter calcoaceticus-Acinetobacter baumannii* complex isolates determined by the broth microdilution method

Isolates	MIC (μg/mL)	
	Zosurabalpin	Cefiderocol
Carbapenem-susceptible *Acinetobacter nosocomialis* (*n* = 10)		
CS-17	0.125	0.03
CS-20	0.125	0.12
CS-22	0.125	0.5
CS-23	1	0.5
CS-25	0.125	0.5
CS-26	0.5	0.25
CS-27	0.015	0.5
CS-28	0.25	0.25
CS-29	0.25	0.25
CS-30	0.25	0.25
Carbapenem-susceptible *Acinetobacter seifertii* (*n* = 1)		
CS-24	0.125	0.5
Carbapenem-susceptible *Acinetobacter pittii* (*n* = 1)		
CS-5	0.125	0.5
Carbapenem-susceptible *Acinetobacter lactucae* (*n* = 1)		
CS-19	1	0.5

## DISCUSSION

In this study, we evaluated the *in vitro* activity of ZAB and several newly developed antimicrobial agents against 206 *A. baumannii* clinical isolates. ZAB demonstrated excellent activity against both CSAB and CRAB, with MIC_90_ values of 0.25 and 1 μg/mL, respectively. Notably, ZAB maintained potent activity against CRAB isolates that were resistant to FDC or COL, with MIC_90_ values of 1 and 2 μg/mL, respectively. Importantly, for the CRAB isolate resistant to both FDC and COL, the MIC of ZAB was only 0.5 μg/mL.

To date, reports on the activity of ZAB against clinical *A. baumannii* isolates remain limited. Hawser et al. reported MIC_50_/MIC_90_ values of 0.25/0.5 μg/mL for ZAB against 83 carbapenem-resistant ACB complex isolates (24), which are comparable to our findings. Another study that included 1,575 ACB complex isolates demonstrated that 99.7% of CR ACB complex isolates were inhibited by ZAB at concentrations ≤ 1 µg/mL (25). In our study, 95.8% of CRAB isolates were inhibited at the same concentration threshold. These data collectively indicate that ZAB exhibits consistently potent *in vitro* activity against clinical CRAB isolates across different strain collections. The two studies discussed above evaluated ZAB activity using collections of ACB complex isolates rather than *A. baumannii* specifically. Although ZAB has previously been studied in *A. baumannii* through *in vitro* and *in vivo* killing-kinetics experiments, those studies did not perform MIC testing (26). To our knowledge, the present work is the first to evaluate the *in vitro* activity of ZAB against *A. baumannii* based on MIC testing.

In this study, we identified eight CRAB isolates with ZAB MICs greater than 1 μg/mL (six isolates with MICs of 2 μg/mL and one isolate each with MICs of 4 and 8 μg/mL). Previous work has shown that exposure to 2×, 4×, 8×, and 16 × MIC can induce spontaneous mutation frequencies ranging from 10^−6^ to 10^−9^, with resulting MIC increases of up to 64-fold (27). Among the mutants that emerged under these conditions, substitutions have been detected in components of the LptB_2_FGC LPS transporter, including the LptF and LptG subunits, as well as in LpxM, which participates in LPS biosynthesis, and in the AdeRS two-component regulatory system involved in efflux regulation. Most mutations were point mutations, and a smaller number were caused by the insertion of IS elements. In the induced mutant strains, several point mutations were identified, including LptF E249K, W271S, I317L, A318E, K320Q, R322L, R322C, and I323K, as well as LptG G40D. As ZAB interacts with both LptF/G and LPS (16), mutations at these positions may alter the interactions required for ZAB binding and reduce its activity (27). LpxM participates in the hepta-acylation of lipid A, and mutations in LpxM result in the production of penta-acylated LPS (27). Such underacylated LPS structures may weaken ZAB–LPS interactions or reduce the efficiency with which ZAB blocks LPS translocation. Mutations in AdeRS, which regulates the AdeABC efflux system (28), may also lead to its upregulation, thereby enhancing ZAB efflux and diminishing intracellular drug accumulation. In our study, the isolates with higher ZAB MICs were naturally occurring clinical isolates rather than laboratory-induced mutants. The mechanisms underlying their reduced susceptibility are still unknown, and further investigations, such as whole-genome sequencing, will be necessary to clarify their genetic basis.

In a multinational survey of isolates collected between 2016 and 2021, the average rate of COL resistance among *A. baumannii* was 3.8%, and the rate among CRAB isolates was 5.1% (29). In Asia, COL resistance in *A. baumannii* isolates in 2021 was only 0.8%. Previous reports from Taiwan have shown COL resistance rates of 10.4% in 2007 and 10.1% in 2016 (29, 30). In our study, the proportion of COL-resistant CRAB isolates was 6.3% (Table 3), which was lower than that in earlier reports from Taiwan. We also found that CR-*A. seifertii* exhibited a markedly higher rate of colistin resistance, with 50% (2/4) of isolates being resistant. A prior investigation similarly noted that only 39.8% of *A. seifertii* isolates were susceptible to colistin when compared with 79.1% of *A. nosocomialis* (31). In our collection, CR-*A. seifertii* isolates also showed relatively high FDC MICs, with MICs ranging from 2 to 16 μg/mL (Table 5). In contrast, ZAB MICs for these isolates ranged from 0.06 to 0.5 μg/mL, suggesting that ZAB may hold promise for treating COL-resistant *A. seifertii* infections (Table 5).

FDC is considered one of the most promising newly developed agents for treating carbapenem-resistant gram-negative bacteria (32). Although its susceptibility rates are generally lower than those of COL, FDC can be a preferred option in patients with impaired renal function, in whom colistin use is limited (32). However, several studies have already reported substantial rates of FDC resistance among CRAB isolates. For example, Smoke et al. and Piccica et al. reported resistance rates ranging from 35.7 to 60% (33, 34). In our study, the proportion of FDC-resistant CRAB isolates was 11.1% (Table 3). Despite being regarded as one of the few remaining therapeutic options for CRAB (35), FDC resistance remains a significant concern, particularly as our study identified CRAB isolates that were resistant to both FDC and COL.

Eravacycline is a novel synthetic fluorocycline that has been approved by both the U.S. Food and Drug Administration (FDA) and the European Medicines Agency (EMA) for the treatment of complicated intra-abdominal infections (cIAIs) in adults (36). Current evidence indicates that eravacycline achieves favorable clinical cure rates in *A. baumannii* cIAIs, making it a valuable therapeutic option for these infections (37). Although breakpoints for eravacycline against *A. baumannii* have not yet been established, previous studies have reported eravacycline MIC_50_/MIC_90_ values of approximately 0.25–0.5/1 μg/mL for CRAB, which is consistent with our findings (0.5/1 μg/mL) (Table 2; Fig. 1) (38–40). Based on the tentative epidemiological cut-off value (ECOFF) proposed by Jing et al., using ≤0.25 mg/L as the susceptibility threshold (41), 18.5% of the CRAB isolates in our study would be considered susceptible (Fig. 1). Using the PK/PD breakpoints recommended by CASFM, in which ≤0.5 mg/L is considered susceptible (40), the susceptibility rate increases to 74.1% (Fig. 1). Although the MIC90 of eravacycline in our study was comparable to that of ZAB, when susceptibility is interpreted according to the currently proposed thresholds (≤1 mg/L for ZAB), the susceptibility rate of CRAB to ZAB (95.8%) was higher than that to eravacycline (Fig. 1).

This study has several limitations. First, the numbers of CSAB and non-*baumannii* ACB isolates were relatively small, which may limit the accuracy of MIC-related statistical estimates for these groups. Second, molecular typing was not performed, and we were, therefore, unable to determine whether the isolates belonged to specific lineages that might have influenced their susceptibility profiles.

In conclusion, we demonstrated that ZAB exhibits potent *in vitro* activity against CRAB, CSAB, and non-*baumannii* ACB complex isolates. Notably, ZAB remained active against CRAB isolates that were resistant to FDC, COL, or both agents.

## Data Availability

The data sets used during the current study are available from the corresponding author on reasonable request.

## References

[1] Zhang S, Di L, Qi Y, Qian X, Wang S. 2024. Treatment of infections caused by carbapenem-resistant *Acinetobacter baumannii*. Front Cell Infect Microbiol 14:1395260. doi:10.3389/fcimb.2024.1395260 PMC11287075 39081869

[2] Bazzi W, Abou Fayad AG, Nasser A, Haraoui LP, Dewachi O, Abou-Sitta G, Nguyen VK, Abara A, Karah N, Landecker H, Knapp C, McEvoy MM, Zaman MH, Higgins PG, Matar GM. 2020. Heavy metal toxicity in armed conflicts potentiates AMR in *A. baumannii* by selecting for antibiotic and heavy metal co-resistance mechanisms. Front Microbiol 11:68. doi:10.3389/fmicb.2020.00068 PMC7008767 32117111

[3] Doi Y, Murray GL, Peleg AY. 2015. *Acinetobacter baumannii*: evolution of antimicrobial resistance-treatment options. Semin Respir Crit Care Med 36:85–98. doi:10.1055/s-0034-1398388 PMC4465586 25643273

[4] Wong D, Nielsen TB, Bonomo RA, Pantapalangkoor P, Luna B, Spellberg B. 2017. Clinical and pathophysiological overview of *Acinetobacter* infections: a century of challenges. Clin Microbiol Rev 30:409–447. doi:10.1128/CMR.00058-16 PMC5217799 27974412

[5] Dubey V, Reza N, Hope W. 2025. Drug-resistant *Acinetobacter baumannii*: mortality, emerging treatments, and future pharmacological targets for a WHO priority pathogen. Clin Microbiol Rev 38:e0027924. doi:10.1128/cmr.00279-24 PMC12424317 40787933

[6] WHO. 2024. Bacterial priority pathogens list, p 12–13. World Health Organization, Geneva.

[7] El-Sayed Ahmed M-G, Zhong L-L, Shen C, Yang Y, Doi Y, Tian G-B. 2020. Colistin and its role in the Era of antibiotic resistance: an extended review (2000–2019). Emerg Microbes Infect 9:868–885. doi:10.1080/22221751.2020.1754133 PMC7241451 32284036

[8] Landman D, Georgescu C, Martin DA, Quale J. 2008. Polymyxins revisited. Clin Microbiol Rev 21:449–465. doi:10.1128/CMR.00006-08 PMC2493081 18625681

[9] Cai Y, Chai D, Wang R, Liang B, Bai N. 2012. Colistin resistance of *Acinetobacter baumannii*: clinical reports, mechanisms and antimicrobial strategies. J Antimicrob Chemother 67:1607–1615. doi:10.1093/jac/dks084 22441575

[10] Portsmouth S, van Veenhuyzen D, Echols R, Machida M, Ferreira JCA, Ariyasu M, Tenke P, Nagata TD. 2018. Cefiderocol versus imipenem-cilastatin for the treatment of complicated urinary tract infections caused by Gram-negative uropathogens: a phase 2, randomised, double-blind, non-inferiority trial. Lancet Infect Dis 18:1319–1328. doi:10.1016/S1473-3099(18)30554-1 30509675

[11] Wunderink RG, Matsunaga Y, Ariyasu M, Clevenbergh P, Echols R, Kaye KS, Kollef M, Menon A, Pogue JM, Shorr AF, Timsit JF, Zeitlinger M, Nagata TD. 2021. Cefiderocol versus high-dose, extended-infusion meropenem for the treatment of Gram-negative nosocomial pneumonia (APEKS-NP): a randomised, double-blind, phase 3, non-inferiority trial. Lancet Infect Dis 21:213–225. doi:10.1016/S1473-3099(20)30731-3 33058798

[12] Sato T, Yamawaki K. 2019. Cefiderocol: discovery, chemistry, and in vivo profiles of a novel siderophore cephalosporin. Clin Infect Dis 69:S538–S543. doi:10.1093/cid/ciz826 PMC6853759 31724047

[13] Karakonstantis S, Rousaki M, Kritsotakis EI. 2022. Cefiderocol: systematic review of mechanisms of resistance, heteroresistance and *in vivo* emergence of resistance. Antibiotics (Basel) 11:723. doi:10.3390/antibiotics11060723 PMC9220290 35740130

[14] Malik S, Kaminski M, Landman D, Quale J. 2020. Cefiderocol resistance in *Acinetobacter baumannii*: roles of β-lactamases, siderophore receptors, and penicillin binding protein 3. Antimicrob Agents Chemother 64:e01221–20. doi:10.1128/AAC.01221-20 PMC7577126 32868330

[15] Zampaloni C, Mattei P, Bleicher K, Winther L, Thäte C, Bucher C, Adam J-M, Alanine A, Amrein KE, Baidin V, et al.. 2024. A novel antibiotic class targeting the lipopolysaccharide transporter. Nature 625:566–571. doi:10.1038/s41586-023-06873-0 PMC10794144 38172634

[16] Pahil KS, Gilman MSA, Baidin V, Clairfeuille T, Mattei P, Bieniossek C, Dey F, Muri D, Baettig R, Lobritz M, Bradley K, Kruse AC, Kahne D. 2024. A new antibiotic traps lipopolysaccharide in its intermembrane transporter. Nature 625:572–577. doi:10.1038/s41586-023-06799-7 PMC10794137 38172635

[17] Narita S, Tokuda H. 2009. Biochemical characterization of an ABC transporter LptBFGC complex required for the outer membrane sorting of lipopolysaccharides. FEBS Lett 583:2160–2164. doi:10.1016/j.febslet.2009.05.051 19500581

[18] Sperandeo P, Martorana AM, Polissi A. 2017. The lipopolysaccharide transport (Lpt) machinery: a nonconventional transporter for lipopolysaccharide assembly at the outer membrane of Gram-negative bacteria. J Biol Chem 292:17981–17990. doi:10.1074/jbc.R117.802512 PMC5672026 28878019

[19] Owens TW, Taylor RJ, Pahil KS, Bertani BR, Ruiz N, Kruse AC, Kahne D. 2019. Structural basis of unidirectional export of lipopolysaccharide to the cell surface. Nature 567:550–553. doi:10.1038/s41586-019-1039-0 PMC6629255 30894747

[20] CLSI. 2025. Performance standards for antimicrobial susceptibility testing; 35th informational supplement. CLSI Document M100-S35. Clinical and Laboratory Standards Institute, Wayne, PA.

[21] CLSI. 2024. Methods for dilution antimicrobial susceptibility tests for bacteria that grow aerobically. CLSI Document M07-Ed12. Clinical and Laboratory Standards Institute, Wayne, PA.

[22] Lin Y-T, Lin H-H, Chen C-H, Tseng K-H, Ho M-W, Hsueh P-R. 2026. Interpretive agreement of susceptibility between broth microdilution and disk diffusion methods for cefiderocol, using criteria from the clinical and laboratory standards Institute, European committee on antimicrobial susceptibility testing, and the food and drug administration. J Clin Microbiol 64:e0125525. doi:10.1128/jcm.01255-25 PMC12802216 41369533

[23] Hamilton-Miller JMT. 1991. Calculating MIC_50_. J Antimicrob Chemother 27:863–864. doi:10.1093/jac/27.6.863 1938695

[24] Hawser S, Kothari N, Valmont T, Louvel S, Zampaloni C. 2023. 2131. Activity of the novel antibiotic zosurabalpin (RG6006) against Clinical *Acinetobacter* Isolates from China. Open Forum Infect Dis 10. doi:10.1093/ofid/ofad500.1754

[25] Maher J, Castanheira M, Fedler KA, Louvel S. 2026. Activity of zosurabalpin and comparator antimicrobial agents tested against acinetobacter baumannii-calcoaceticus complex (ABC) isolates collected worldwide in 2024. Open Forum Infect Dis 13. doi:10.1093/ofid/ofaf695.1521

[26] Erbetti I, Ferrari L, Ortombina A, Savoia P, Felici A, Bissantz C, Zampaloni C. 2023. *In vitro* and *in vivo* killing kinetics of zosurabalpin (RG6006) against *Acinetobacter baumannii*. Open Forum Infect Dis 10. doi:10.1093/ofid/ofad500.1733

[27] Trauner A, Ortombina A, Erbetti I, Dylus D, Dey F, Leipner M, Scharf S, Misson P, Louvel S, Mattei P, Felici A, Zampaloni C. 2024. Mechanism of in vitro resistance to zosurabalpin (RG6006) in Acinetobacter baumannii, abstr ECCMID Global 2024. Barcelona, Spain.

[28] Marchand I, Damier-Piolle L, Courvalin P, Lambert T. 2004. Expression of the RND-type efflux pump AdeABC in *Acinetobacter baumannii* is regulated by the AdeRS two-component system. Antimicrob Agents Chemother 48:3298–3304. doi:10.1128/AAC.48.9.3298-3304.2004 PMC514774 15328088

[29] Chang KC, Lin MF, Lin NT, Wu WJ, Kuo HY, Lin TY, Yang TL, Chen YC, Liou ML. 2012. Clonal spread of multidrug-resistant *Acinetobacter baumannii* in eastern Taiwan. J Microbiol Immunol Infect 45:37–42. doi:10.1016/j.jmii.2011.09.019 22154678

[30] Lai C-C, Chen Y-S, Lee N-Y, Tang H-J, Lee SS-J, Lin C-F, Lu P-L, Wu J-J, Ko W-C, Lee W-S, Hsueh P-R. 2019. Susceptibility rates of clinically important bacteria collected from intensive care units against colistin, carbapenems, and other comparative agents: results from surveillance of multicenter antimicrobial resistance in Taiwan (SMART). Infect Drug Resist 12:627–640. doi:10.2147/IDR.S194482 PMC6421902 30936726

[31] Lee Y-T, Sun J-R, Li L-H, Yang Y-S, Chang H-M, Lin P-Y, Liao P-H, Kang F-Y, ACTION study group, Chen T-L, Wang Y-C. 2026. Comparison of clinical manifestations, antimicrobial susceptibility patterns, and carbapenem resistance determinants between *Acinetobacter seifertii* and *Acinetobacter nosocomialis* isolated in Taiwan. J Microbiol Immunol Infect 59:351–357. doi:10.1016/j.jmii.2025.08.025 40915961

[32] Yousefi B, Kashanipoor S, Mazaheri P, Alibabaei F, Babaeizad A, Asli S, Mohammadi S, Gorgin AH, Alipour T, Oksenych V, Eslami M. 2024. Cefiderocol in combating carbapenem-resistant *Acinetobacter baumannii*: action and resistance. Biomedicines 12:2532. doi:10.3390/biomedicines12112532 PMC11592056 39595098

[33] Smoke SM, Brophy A, Reveron S, Iovleva A, Kline EG, Marano M, Miller LP, Shields RK. 2023. Evolution and transmission of cefiderocol-resistant *Acinetobacter baumannii* during an outbreak in the burn intensive care unit. Clin Infect Dis 76:e1261–e1265. doi:10.1093/cid/ciac647 PMC10169418 35974429

[34] Piccica M, Spinicci M, Botta A, Bianco V, Lagi F, Graziani L, Faragona A, Parrella R, Giani T, Bartolini A, Morroni G, Bernardo M, Rossolini GM, Tavio M, Giacometti A, Bartoloni A. 2023. Cefiderocol use for the treatment of infections by carbapenem-resistant Gram-negative bacteria: an Italian multicentre real-life experience. J Antimicrob Chemother 78:2752–2761. doi:10.1093/jac/dkad298 PMC10631827 37807834

[35] Serapide F, Guastalegname M, Gullì SP, Lionello R, Bruni A, Garofalo E, Longhini F, Trecarichi EM, Russo A. 2024. Antibiotic treatment of carbapenem-resistant *Acinetobacter baumannii* infections in view of the newly developed β-lactams: a narrative review of the existing evidence. Antibiotics (Basel) 13:506. doi:10.3390/antibiotics13060506 PMC11201171 38927173

[36] Scott LJ. 2019. Eravacycline: a review in complicated intra-abdominal infections. Drugs 79:315–324. doi:10.1007/s40265-019-01067-3 PMC6505493 30783960

[37] Marino A, Augello E, Stracquadanio S, Bellanca CM, Cosentino F, Spampinato S, Cantarella G, Bernardini R, Stefani S, Cacopardo B, Nunnari G. 2024. Unveiling the secrets of *Acinetobacter baumannii*: resistance, current treatments, and future innovations. IJMS 25:6814. doi:10.3390/ijms25136814 PMC11241693 38999924

[38] Seifert H, Stefanik D, Sutcliffe JA, Higgins PG. 2018. *In-vitro* activity of the novel fluorocycline eravacycline against carbapenem non-susceptible *Acinetobacter baumannii*. Int J Antimicrob Agents 51:62–64. doi:10.1016/j.ijantimicag.2017.06.022 28705668

[39] Liu Q, Li S, Wang X, Lin Y, Jiang H, Li N. 2025. Comparative evaluation of eravacycline susceptibility testing methods in 587 clinical carbapenem-resistant *Acinetobacter baumannii* isolates: broth microdilution, MIC test strip and disc diffusion. J Antimicrob Chemother 80:439–444. doi:10.1093/jac/dkae426 39568035

[40] Kinet-Poleur A, Bogaerts P, Montesinos I, Huang TD. 2025. Eravacycline: evaluation of susceptibility testing methods and activity against multidrug-resistant *Enterobacterales* and *Acinetobacter*. Eur J Clin Microbiol Infect Dis 44:2771–2778. doi:10.1007/s10096-025-05235-0 40824337

[41] Jing R, Yi QL, Zhuo C, Kang W, Yang QW, Yu YS, Zheng B, Li Y, Hu FP, Yang Y, Lin J, Zhang G, Zhang JJ, Wang T, Li J, Zhuo CY, Li X, Zhu YF, Xu YC. 2024. Establishment of epidemiological cut-off values for eravacycline, against *Escherichia coli*, *Klebsiella pneumoniae*, *Enterobacter cloacae*, *Acinetobacter baumannii* and *Staphylococcus aureus*. J Antimicrob Chemother 79:2246–2250. doi:10.1093/jac/dkae220 PMC11368425 39011845

